# Feasibility of a Novel Mobile C-Reactive Protein–Testing Device Using Gold-Linked Electrochemical Immunoassay: Clinical Performance Study

**DOI:** 10.2196/18782

**Published:** 2020-09-07

**Authors:** Yuko Gondoh-Noda, Mitsuhiro Kometani, Akihiro Nomura, Daisuke Aono, Shigehiro Karashima, Hiromi Ushijima, Eiichi Tamiya, Toshinori Murayama, Takashi Yoneda

**Affiliations:** 1 Department of Clinical Development Kanazawa University Graduate School of Medicine Kanazawa Japan; 2 Division of Endocrinology and Hypertension, Department of Cardiovascular and Internal Medicine Graduate School of Medical Sciences Kanazawa University Kanazawa Japan; 3 Department of Cardiovascular Medicine, Kanazawa University Graduate School of Medical Sciences Innovative Clinical Research Center, Kanazawa University (iCREK) Kanazawa Japan; 4 Innovative Clinical Research Center, Kanazawa University (iCREK) Kanazawa Japan; 5 BioDevice Technology Ltd Nomi Japan; 6 Department of Applied Physics Graduate School of Engineering Osaka University Nomi Japan; 7 Department of Health Promotion and Medicine of the Future Graduate School of Medical Sciences Kanazawa University Kanazawa Japan; 8 Program Management Office for Medical Innovation Graduate School of Medical Sciences Kanazawa University Kanazawa Japan

**Keywords:** gold-linked electrochemical immunoassay (GLEIA), home-based care, mobile CRP testing device, mHealth, diagnostic, infection, assay, CRP, c-reactive protein, immunoassay

## Abstract

**Background:**

Home-based care is one of the most promising solutions to provide sufficient medical care for several older patients in Japan. However, because of insufficient diagnostic devices, it is sometimes difficult to detect early signs of the occurrence or worsening of diseases, such as infections under home-based care settings. C-reactive protein (CRP) is highly sensitive to diagnosing infections, and its elevation can help diagnose acute infection in older patients. Therefore, a CRP-measuring device that can be used in such a specific occasion is needed for home-based care. However, aspects such as its size, weight, and procedure are still challenging with respect to the practical use of mobile devices that quantitatively measure CRP levels easily and quickly under home-based care settings.

**Objective:**

We developed a new mobile, rapid CRP measurement device using a gold-linked electrochemical immunoassay (GLEIA) system. The aim of this study was to evaluate the feasibility of this mobile CRP-testing device.

**Methods:**

First, we assessed the performance of bare GLEIA-based electrode chips as the foundation of the device. After embedding the bare GLEIA-based electrode chips in a special plastic case and developing the mobile CRP-testing device, we further tested the device prototype using clinical blood samples. Finally, we evaluated the intra-assay variability for precision in the same condition and inter-assay variability for reproducibility in different conditions.

**Results:**

Blood samples for analysis were obtained by direct vein puncture from outpatients (N=85; females: 57/85; males: 28/85; age: 19-88 years) at Kanazawa University Hospital in Japan. For performance evaluation of bare GLEIA-based electrode chips, we used 85 clinical blood samples. There was a significant positive correlation between the electrode-predicted CRP levels and the reference CRP concentrations (R^2^=0.947; *P*<.001). The assembled device was mobile (size 45×90×2.4 mm; weight 10 g) and disposable. The minimum volume of the sample needed for measuring CRP was 1.4 µL. The estimated preanalytical time was approximately 7 minutes and 40 seconds, and analysis time was approximately 1 minute and 10 seconds. Subsequently, for performance evaluation of the mobile CRP-testing device using GLEIA-based electrode chips, we used 26 clinical blood samples and found a significant positive correlation between the mobile device-predicted CRP levels and the reference CRP concentrations (R^2^=0.866, *P*<.001). The intra-assay variabilities were 34.2%, 40.8%, and 24.5% for low, medium, and high CRP concentrations, respectively. The inter-assay variabilities were 46.5%, 38.3%, and 64.1% for low, medium, and high CRP concentrations, respectively.

**Conclusions:**

Our findings suggest that this new mobile CRP-testing device might be suitable for use in home-based care settings.

## Introduction

Home-based care is one of the most promising solutions to provide sufficient medical care for the large number of older patients in Japan, because the ageing rate in Japan is expected to reach as high as 26.2% in 2020 [1]. Home-based care mainly provides general medical care for older patients who face difficulties in seeing doctors at hospitals or those who wish to live in their homes rather than at medical facilities. Home-based care also meets the governmental demand in Japan to alleviate hospitalization burden and to decrease total medical costs [2]. However, because of insufficient diagnostic devices, it is sometimes difficult to detect early signs of disease occurrence or worsening such as by infections in these settings [2-4]. Diagnostic delay worsens the prognosis of infectious diseases [5]. Thus, a better tool for early diagnosis of infection is needed in home-based care settings.

Nowadays, blood tests to check systemic inflammation are being used in Japanese hospitals, as they are easy and useful for early diagnosis of infectious diseases [6-8]. C-reactive protein (CRP), a plasma protein and a major component of inflammatory reactions, is widely used as an objective indicator of systemic inflammation [9-11]. CRP is highly sensitive for diagnosing infections [12], and its elevation can help diagnose acute infection in older patients [13]. It is also known that CRP is a useful tool for correlation with longer overall survival in early-stage malignancies [14]. Although at least 8 different semi-quantitative strips and quantitative point-of-care tests for CRP level have already been reported, each of these methods has both practical merits and demerits [15]. One of them is too big and heavy to carry, and the others need long times to obtain the result or have limitations related to their material.

The gold-linked electrochemical immunoassay (GLEIA) system is a highly sensitive electrochemistry assay that uses gold nanoparticle–labeled antibodies [16], and provides advantages in the aspects of miniaturization and time saving. Therefore, we developed a new mobile and rapid CRP measurement device using a GLEIA system for quantitative, easy, and immediate measurement of serum CRP levels from patients’ blood samples. In this study, we evaluated the feasibility of a new mobile, rapid CRP measurement device using the GLEIA system.

## Methods

### Overview

**Figure 1:**
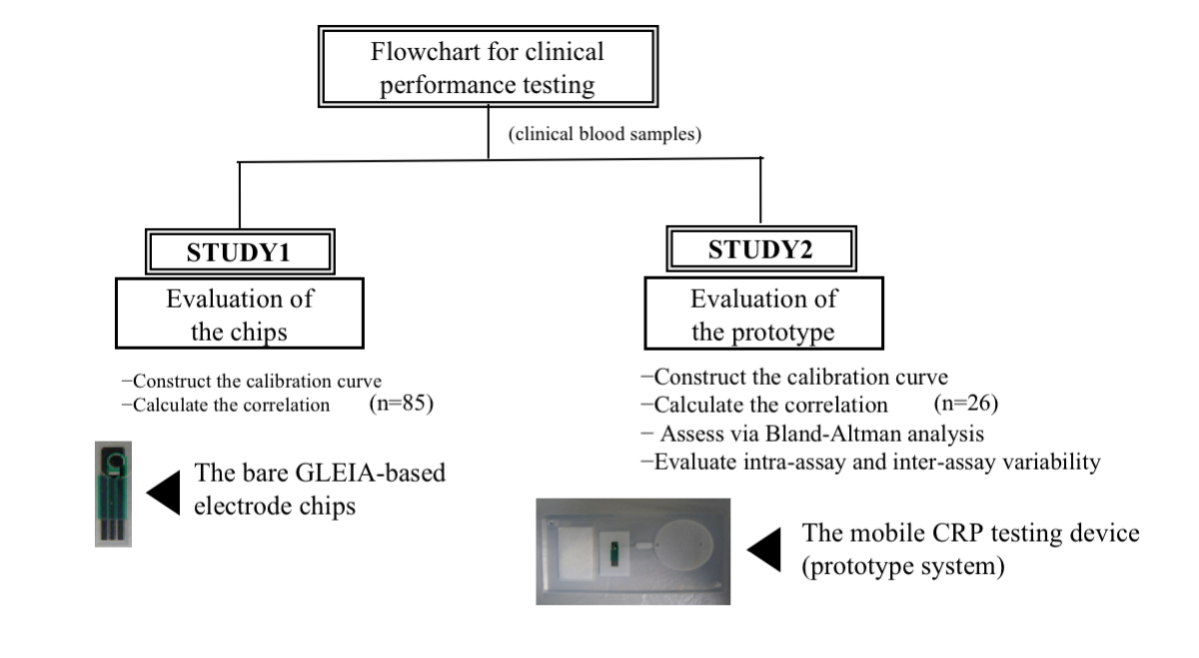
Flowchart of the clinical performance tests. CRP: c-reactive protein; GLEIA: gold-linked electrochemical immunosorbent assay.

Figure 1 shows the flowchart of clinical performance tests for the mobile CRP-testing device.

In study 1, we first assessed the electrolytic currents associated with standardized CRP concentrations (0, 0.25, 1, 4, 8, and 16 mg/dL) using bare GLEIA-based electrode chips and constructed a “current-to-CRP” curve for calibration. Next, we established a prediction formula using the calibration curve to determine the serum CRP concentration from the electrolytic current. We then recruited 85 participants to validate the bare GLEIA-based electrode chips using clinical specimens. The participants underwent a blood test, and their CRP level was checked in advance at Kanazawa University Hospital for suspected infectious diseases or to evaluate the condition of a disease. We calculated the correlation between chip-based measurements and laboratory measurements. After developing the mobile CRP-testing device (prototype system) embedded with the GLEIA-based electrode chips, we repeated the verification procedure using that prototype system (study 2). In study 2, we assessed the electrolytic currents associated with standardized CRP concentrations (0, 0.25, 1, 4, 8,16, and 32 mg/dL) using the prototype device and constructed a “current-to-CRP” curve for calibration. We then validated the prototype system using 25 clinical samples as in study 1. We also performed Bland-Altman analysis and intra- and inter-assay variability testing of the mobile CRP-testing device for practical use.

This study was approved by the Ethics Committees of Kanazawa University (no. 2017078). The written informed consent was waived because we used existing samples with no additional invasion to the patients. We used an opt-out approach to protect the patients’ rights to reject the participate in this study. This was done by posting the study description document on the website of Kanazawa University Hospital, which is open to every patient. All methods were performed in accordance with the approved guidelines and regulations.

### Blood Samples

Blood samples were obtained by direct vein puncture from outpatients (N=85; females: 57/85; male: 28/85; age: 19-88 years) at Kanazawa University Hospital in Japan. Their CRP concentrations ranged from 0.0 to 15.5 mg/dL. These blood samples were collected in blood collection tubes with sodium citrate and were centrifuged at 3000*g* for 10 minutes within 1 day after collection. We used 100 µL of serum from these blood samples for the GLEIA-based measurement of CRP levels. Serum was transferred to microtubes and frozen at –80°C, and it took approximately 1 week before the serum was frozen. Frozen serum samples packed in a dedicated container were properly transported with ice packs to the laboratory of BioDevice Technology Inc by the coinvestigators. Serum samples were thawed and analyzed using the bare GLEIA-based electrode chips and the prototype system.

### The GLEIA System

In the GLEIA method, immobilized primary antibodies on electrodes and gold nanoparticle–linked secondary antibodies form a sandwich structure with the antigen. Anti–C-reactive protein antibody (RRID: AB_2085618, Abcam) and Gold nanoparticles of 60 nm (Gold nanoparticles 60 nm, BBI Solutions) were used in this study. After the reaction, the free gold-linked antibodies were removed by washing with an acidic solution to fix the gold nanoparticles on the electrode surface and to oxidize the gold nanoparticles. After measuring the reduction current of oxidized gold nanoparticles using differential pulse voltammetry (DPV), we quantified the CRP levels using a “current-to-CRP” calibration curve, which was generated using quality control serum samples. We used the equation of the calibration curve to determine the CRP concentrations. The results of the test were not blinded to the operators performing the GLEIA measurements.

All 85 samples were analyzed using GLEIA electrode chips to determine the correlation between CRP levels (measured using the bare GLEIA-based electrode chips) and the laboratory-measured CRP concentrations as reference. Initially, we measured the reduction current; if the current exceeded the maximum value, it was rechecked up to 3 times. Data were rechecked in 7 cases. Some samples were rechecked at least once. Following this rechecking process, 4 samples were excluded from further analyses because no data were obtained.

After developing the new GLEIA measurement system, we used 26 samples for further analysis using the prototype. If the current exceeded the maximum value, the data were rechecked; one sample showed no data.

### Performance Evaluation of the Bare GLEIA-Based Electrode Chips

Initially, serum was added to a microtube containing gold-linked secondary antibodies; these serum samples were then mixed with the diluting solution and diluted 1000-fold. The samples were then placed on the GLEIA electrode chips immobilized primary antibodies, and after formation of the sandwich structure resulting from the antigen-antibody reaction, excess antibodies were washed out by rinsing with the washing solution in a beaker. Finally, we connected the analyzer which uses DPV system to the electrode chips in order to determine the results and calculated the CRP levels using an Internet of Things (IoT) device. This IoT device is a computer terminal using proprietary software for the analyzer (BDTminiSTAT100) and the environment is Windows 7 to 10.

### Performance Evaluation of the Mobile CRP-Testing Device

The mobile device size was 45×90×2.4 mm, and its weight was 10 g. The analyzer (BDTminiSTAT100) was 50×70×25 mm in size, and it weighed 65 g. The adaptation equipment size was 70×120×15 mm, and it weighed 130 g. This adaptation equipment is necessary to connect the electrode to the analyzer in the correct manner. Initially, serum was added to wells coated with the gold-linked antigen, and then serum samples were mixed with the diluting solution and diluted 1000-fold. These samples were then placed on the GLEIA electrode, and after formation of the sandwich structure resulting from the antigen-antibody reaction, excess antibodies were washed out by attaching a liquid tank to the prototype system. Finally, we connected the analyzer to determine the results and calculated the CRP levels using an IoT device for Windows.

### Laboratory Measurements

Results from laboratory measurements were determined before the results from GLEIA measurements. We used Quoligent CRP reagent (Sekisui Medical Co, Ltd) and a Hitachi LABOSPECT-L instrument for laboratory measurements and then visualized the results using electronic medical records within a few hours. The analytical range of the measurements was between 0.02 and 42 mg/dL, and the intra-assay coefficient of variation was less than or equal to 5%.

### Statistical Analysis

Data were expressed as means (SD). Statistical analyses were performed using Excel 2016 (Microsoft) with an add-in software Statcel4 (OMS). Spearman rank correlation coefficient was used to analyze correlations between GLEIA measurements and laboratory measurements, and *P* values less than 0.05 were considered statistically significant. Bland-Altman plots were drawn using R version 3.4.3.

## Results

**Figure 2:**
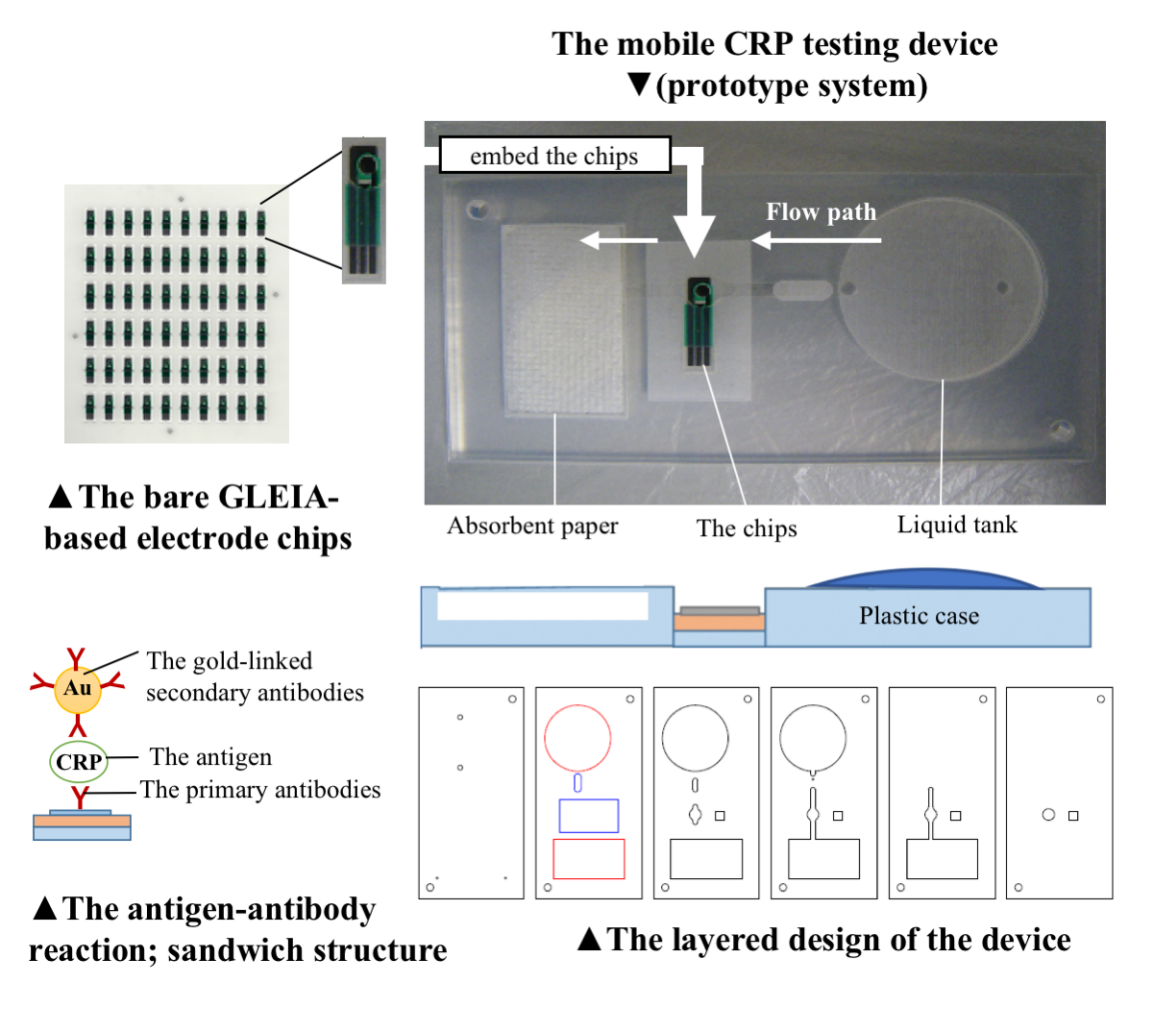
Production process from the chips to the prototype and the schematic of GLEIA measurement. CRP: c-reactive protein; GLEIA: gold-linked electrochemical immunosorbent assay.

The production process from the chips to the prototype and the diagram depicting the reaction occurring on the chip are shown in Figure 2. The mobile CRP-testing device (prototype system) was embedded with the GLEIA-based electrode chips and the liquid tank. The diagram depicting the reaction occurring on the chip is shown at the lower left. The cross-section of plastic case is shown at the lower right.

### Study 1: Performance of the Bare GLEIA-Based Electrode Chips

**Figure 3:**
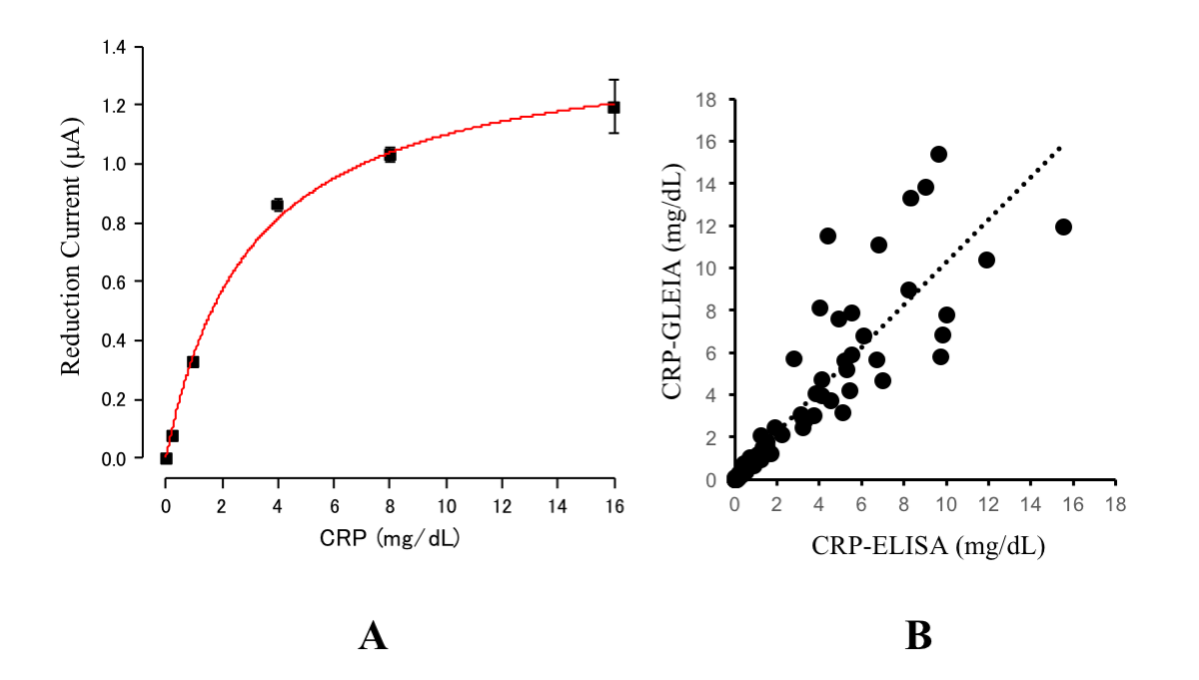
The current-to-CRP calibration curve (A) and the correlation between the chips-measured CRP levels and the laboratory-measured CRP levels (B). CRP: c-reactive protein; ELISA: enzyme-linked immunosorbent assay.

First, we tested the performance of bare GLEIA-based electrode chips. We constructed an electrolytic reduction current-to-CRP calibration curve based on the electrolytic current produced by the chips and each standardized CRP concentration using the Michaelis-Menten model [17]. Figure 3 A represents the calibration curve between reduction current (μA) and CRP (mg/dL) level measured using GLEIA electrode chips. The formula for the Michaelis-Menten model between the current (μA) and CRP (mg/dL) was as follows: y = V_max_*x / (K_m_ + x), V_max_ = 1.43697, K_m_ = 30.56623.

For performance evaluation, we measured the reduction current using 85 clinical blood samples. If the current exceeded the maximum value, it was rechecked at least once because it did not fit within the calibration curve. Following this rechecking process, 4 samples were excluded from further analyses because no data were obtained. Then, we tested the correlation between the electrode-predicted CRP levels (based on the prediction formula) and the laboratory-measured CRP concentrations (used as reference). Figure 3 B presents the correlation between CRP levels measured using the GLEIA electrode chips and the laboratory-measured CRP concentrations (reference). There was a significant positive correlation (R=0.972962; n=81; y=1.0049 + 0.2305x; *P*<.001) between the CRP levels detected by these 2 methods. There was a significant positive correlation between the electrode-predicted CRP levels and the reference CRP concentrations (*R*^2^=0.947; *P*<.001).

### Study 2: Performance of the Mobile CRP-Testing Device Using the GLEIA-Based Electrode Chips

Next, we assessed the performance of the mobile CRP-testing device using the GLEIA-based electrode chips tested in study 1. The device was mobile (size 45×90×2.4 mm; weight 10 g) and disposable; and the adaptation equipment could be used several times. The minimum amount of material required for measuring CRP was 1.4 µL. The estimated preanalytical time was approximately 7 minutes and 40 seconds, and the analysis time was approximately 1 minute and 10 seconds.

**Figure 4:**
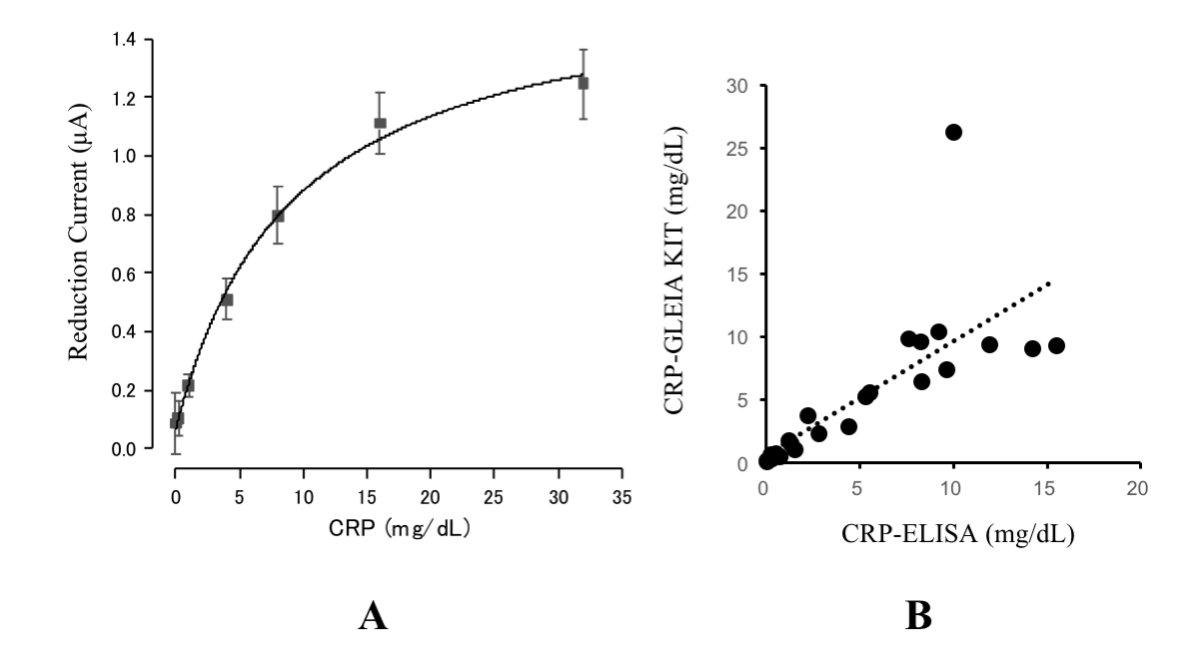
The current-to-CRP calibration curve (A) and the correlation between the prototype-measured CRP levels and the laboratory-measured CRP levels (B). CRP: c-reactive protein; ELISA: enzyme-linked immunosorbent assay.

We again constructed an electrolytic reduction current-to-CRP calibration curve between the electrolytic current produced by the mobile CRP-testing device and each standardized CRP concentration using the Michaelis-Menten model. Figure 4 A presents the calibration curve between the reduction current (μA) and CRP (mg/dL) using the prototype mobile CRP-testing device. The CRP level prediction formula based on the calibration curve was as follows: y = V_max_*x / (K_m_ + x), V_max_ = 1.56341, Km = 74.54069.

For performance evaluation, we measured the reduction current using 26 clinical blood samples. Of these, 1 sample yielded no data and was excluded from further analyses. We then tested the correlation between electrode-predicted CRP levels using the prediction formula and the laboratory-measured CRP concentrations as a reference. Figure 4 B presents Correlation between the mobile CRP-testing device and the laboratory-measured CRP concentrations (reference). There was a significant positive correlation (R=0.930769; n=25; y=0.898x + 0.6919; *P*<.001) between the CRP levels detected by these 2 methods. As expected, we found a significant positive correlation between the mobile device-predicted CRP levels and the reference CRP concentrations (*R*^2^=0.866, *P*<.001).

**Figure 5:**
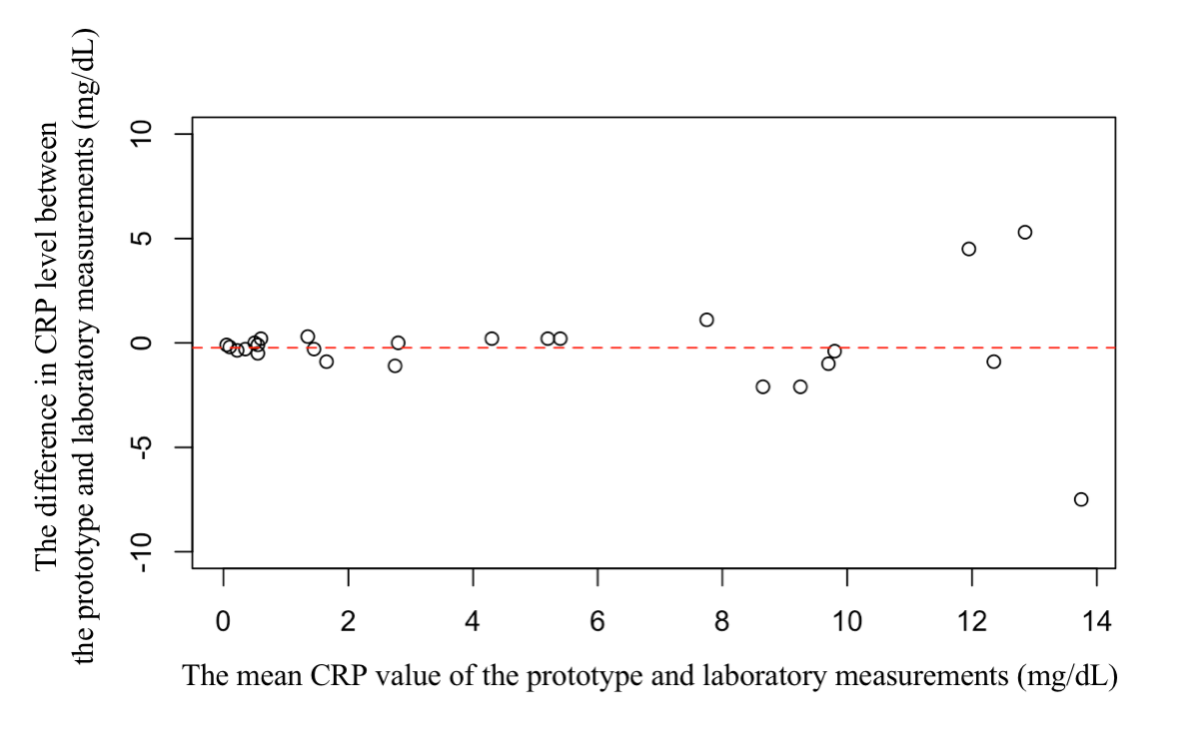
Bland-Altman plot between GLEIA measurement and the laboratory-measured CRP concentrations (reference). CRP: c-reactive protein.

Furthermore, we performed Bland-Altman analysis for elucidating whether there were any systematic errors. The results of the agreement between the CRP level using the prototype and laboratory measurements are shown graphically in Figure 5. Differences between the CRP levels measured using the mobile CRP-testing device and the laboratory-measured CRP concentrations were calculated for each method and were plotted against the mean values of both measurements. The mean difference was 0.234. On an average, higher values (CRP > 10 mg/dL) tended to exhibit greater discrepancies between the values compared to the lower values.

### Intra- and Inter-Assay Variability Assessments

Finally, we evaluated the (1) intra-assay variability for precision in the same condition and (2) inter-assay variability for reproducibility in different conditions. First, intra-assay variability of the prototype system was assessed by determining the coefficient of variation using 5 replicate measurements for each of the following 3 samples (low, medium, and high CRP concentration levels) on the same day and in the same conditions. These 3 samples were selected from the rest of the above 85 specimens in terms of CRP concentration for matching approximately low (0 mg/dL), medium (5 mg/dL), and high (over 10 mg/dL) CRP concentration levels, respectively. The intra-assay variabilities were 34.2% for the low (mean 0.5 mg/dL), 40.8% for the medium (5.1 mg/dL), and 29.5% for the high CRP concentrations (14.7 mg/dL).

Second, inter-assay variability of the prototype system was assessed by determining the coefficient of variation using 15 replicate measurements for each of the above 3 samples (low, medium, and high CRP concentration levels) in different conditions. We checked the same sample 3-times on 5 days with different technicians and devices (miniSTAT/pipette). The inter-assay variabilities were 46.5% for the low (mean 0.5 mg/dL), 38.3% for the medium (5.1 mg/dL), and 64.1% for the high CRP concentrations (14.7 mg/dL).

## Discussion

### Principal Results

In this study, we developed and evaluated the feasibility of a new mobile rapid CRP measurement device using the GLEIA system. Comparison with conventional measurement using enzyme-linked immunosorbent assay (ELISA) has often been performed to evaluate new devices [18]. Therefore, we first assessed the performance of bare GLEIA-based electrode chips and found a significant positive correlation between the electrode-predicted CRP levels and the reference CRP concentrations. Next, we assembled and tested the mobile CRP-testing device (prototype system) embedded with the GLEIA-based electrode and found a significant positive correlation between the mobile device-predicted CRP levels and the reference CRP concentrations. We also found the limitation of increasing discrepancy at higher levels of CRP. There is a possibility to reduce the discrepancy by optimizing the dilution of the samples. Further studies are needed to use this new mobile device for quantitative measurement of CRP levels easily and quickly in home-based care settings.

This study yielded several results. The GLEIA-based electrode chip was feasible for measuring CRP levels using clinical specimens. Initially, the GLEIA-based electrode chip was mass-produced in a sheet form. Therefore, we generated calibration curves for each measurement to avoid differences between electrodes. As these electrodes are disposable, there is no need to worry about contamination; and their flat shape makes it easy to modify and fix them to a device. This GLEIA system measures the reduction current of oxidized gold nanoparticles, which forms a sandwich structure with the antigen, through an antigen-antibody reaction; here, we quantified CRP levels using a “current-to-CRP” calibration curve. Thus, it takes a short time to measure the sample and the analyzer needs not to be large. We guaranteed the performance of the bare GLEIA-based electrode chips, which serve as a foundation for the mobile device. However, for the measurement procedure, we had to prepare a washing solution to remove the excess labeled antibody after the antigen-antibody reaction. We performed a rinsing step using this solution when the bare GLEIA-based electrode chips alone were used. This process is difficult in home-based care settings. Hence, we combined the GLEIA original electrode chips and a liquid tank to simplify the procedure. As a result, the rinsing step is completed within the device; the solution is pushed out through the flow path to wash out the electrodes and is imbibed by the equipped absorbent paper. This avoids the need for additional equipment and reduces the risk of contamination. The combined device was compact and lightweight, and similar in size to a name card. To our knowledge, such a lightweight device has not been reported yet [14]. Moreover, this system (patent pending) reduces the necessity for complicated procedures and avoids the risk of mistakes during artificial manipulation. However, quality assurance and training protocols need to be established to ensure maximal benefits for patient care and efficiency [19].

Further, our novel mobile CRP detector using the GLEIA-based electrode chip system was also feasible for measuring serum CRP levels. After the foundation of the device was prepared using plastic, the liquid tank was filled with the washing solution, and the device was ready to use, we confirmed that the CRP levels measured using new mobile rapid CRP-testing device also showed a significant positive correlation with the reference CRP concentrations. We then connected this device with an analyzer connected to a PC for visualizing the results on a PC screen. Thus, this device was also applicable for use with the IoT approach [20]. Alternatively, the results could be observed on a smart device if a Bluetooth-compatible analyzer were used simultaneously. This approach may be useful for data storage, management, and telemedicine, allowing us to share data with a physician at a distant location.

### Limitations

This study has some limitations. First, we investigated GLEIA using clinical specimens that were already subjected to centrifugal separation in a laboratory. In home-based care settings, we hope to be able to perform the test with a simple fingertip prick, rather than with serum samples obtained after centrifugation. Second, detection of high CRP levels by the device resulted in more dramatic differences between 2 measurements than in the case of low CRP levels, and some cases needed retesting or were unmeasurable. This may be due to the use of a calibration curve that plateaus at high CRP levels. All 4 samples, which had no data in this study, showed a reduction current over 1.2 μA; and the reference CRP levels using ELISA for these 4 samples were 9.2, 13.3, 7.6, and 14.2 mg/dL, respectively. We calculated the correlation without these unmeasurable samples. Therefore, we have to mention that this correlation does not focus on high ranges of CRP level. We thus have to avoid situations wherein results showing high CRP levels may not be accurate and could therefore result in incorrect clinical decisions. Given the nature of this cause, further improvement of this approach could be theoretically achieved by optimizing the sample dilution. Using the current methods, the serum samples were mixed with the diluting solution and diluted 1000-fold. However, if they are diluted up to 10,000-fold, the calibration curve cannot form a plateau even at a high CRP range, thus theoretically reducing errors. There is no data from a similar mobile CRP-testing device for comparison; therefore, we need to improve the results of intra- and inter-assay variabilities using the same solution. However, in home-based care settings, cases with CRP levels over 4.35 mg/dL and those with suspected pneumonia [21] (and exceeding this level) need prompt medical attention. Thus, there is a possibility to contrive the unmeasurable limitation as an indication of “extremely high CRP level.”

In this study, we established a new mobile rapid CRP-testing device using a GLEIA system. There was a significant correlation between the CRP levels measured using the new mobile CRP-testing device and the laboratory-measured CRP concentrations. Because of its portability, we consider that the device might be suitable for use in home-based care settings.

### Comparison With Prior Work

To date, at least 9 different types of semiquantitative strips and quantitative point-of-care testing devices have been developed for measuring CRP levels; these approaches include immunochromatographic assays, immunoturbidimetric assays, solid-phase immunochemical assays, and vertical flow assays with 3D paper-based microfluidics [22]. In some products, the analyzer weights ranged from 1.7 to 35 kg. These products were not suitable to be carried and used in home-based care settings, and other assays had limitations in their clinical use.

### Conclusions

The GLEIA-based mobile device developed in this study allows quantitative measurement of serum CRP levels from patients’ blood samples easily and immediately. Our findings indicate that this new mobile CRP-testing device could be suitable for use in home-based care settings. Further research is needed to apply this device to more user-friendly device or multi-item measurement.

## Abbreviations

CRP: C-reactive protein
DPV: differential pulse voltammetry
ELISA: enzyme-linked immunosorbent assay
GLEIA: gold-linked electrochemical immunoassay
IoT: Internet of Things
